# Human Metapneumovirus Infection Inhibits Cathelicidin Antimicrobial Peptide Expression in Human Macrophages

**DOI:** 10.3389/fimmu.2018.00902

**Published:** 2018-05-04

**Authors:** Youxian Li, Stine Østerhus, Ingvild B. Johnsen

**Affiliations:** Department of Clinical and Molecular Medicine, Faculty of Medicine and Health Sciences, Norwegian University of Science and Technology, Trondheim, Norway

**Keywords:** human metapneumovirus, human macrophages, cathelicidin, vitamin D signaling, C/EBPα

## Abstract

Human cathelicidin antimicriobial peptide (CAMP) is a critical component of host innate immunity with both antimicrobial and immunomodulatory functions. Several pathogens have been shown to downregulate CAMP expression, yet it is unclear if such modulation occurs during a viral infection. In this study, we showed that infection with human metapneumovirus (hMPV), one of the leading causes of respiratory tract infections in young children, strongly suppressed basal and vitamin-D induced CAMP expression in human macrophages. hMPV-mediated suppression of CAMP did not correlate with reduced transcriptional expression of key vitamin D signaling components, such as CYP27B1 or vitamin D receptor, suggesting a vitamin D-independent mechanism. Blocking interferon-signaling pathways did not reverse hMVP-mediated suppression of CAMP, indicating that the suppressive effect is largely interferon-independent. Instead, we identified C/EBPα as the key modulator of hMPV-mediated suppression of CAMP. hMPV infection strongly repressed the expression of C/EBPα, and a knockdown study confirmed that C/EBPα is critical for CAMP expression in human macrophages. Such modulation of CAMP (and C/EBPα) could be reproduced by TLR1/2 ligand treatment in human macrophages, suggesting a common mechanism underlying pathogen-mediated downregulation of CAMP through C/EBPα. This study opens up a new understanding of altered human antimicrobial responses following infections.

## Introduction

Antimicrobial peptides (AMPs), or host defense peptides, are a conserved component of natural defenses in all complex life forms (1). AMPs are typically short peptides containing abundant positively charged and hydrophobic residues. Originally studied for their direct antimicrobial activities, AMPs were later shown to have many other immunomodulatory functions (1, 2). Numerous AMPs have been identified in humans, notably histatins, defensins, and cathelicidin [cathelicidin antimicrobial peptide (CAMP)] (3). Human CAMP gene encodes the full-length 18 kDa precursor, hCAP-18. The precursor contains a conserved cathelin domain at the N-terminal and the functional antimicrobial domain at the C-terminal. Mature CAMP (usually referred to as LL-37) is released by proteolytic cleavage from the precursor mediated by proteinase 3 (4). CAMP is expressed in various cell types, including epithelial cells, adipocytes, as well as immune cells, such as neutrophils and macrophages (4–8). Mature CAMP (LL-37) has been shown to have direct antimicrobial activities against a broad range of pathogens such as Gram positive or negative bacteria, mycobacteria, fungi, and viruses (9, 10). It has also been demonstrated to possess many other modulatory functions, including anti-inflammatory activity by neutralizing endotoxins, direct chemoattractant activities, and wound-healing effects (11–13). Human CAMP gene has a vitamin D response element sequence in its promoter region and vitamin D has been shown to induce CAMP expression (6, 14). Two molecules are of particular importance in the vitamin D signaling pathway: CYP27B1, which metabolizes inactive circulating vitamin D [25(OH)D_3_] into active vitamin D [1,25(OH)_2_D_3_], and vitamin D receptor (VDR), which binds to [1,25(OH)_2_D_3_] and forms a heterodimer with retinoid X receptor to regulate the transcription of numerous vitamin D-targeted genes, including CAMP (15). This vitamin D-mediated antimicrobial response has been demonstrated to be important for protecting monocytes and macrophages from infections with intracellular pathogens, such as *Mycobacterium tuberculosis* (Mtb) (16–18).

Human metapneumovirus (hMPV) is a common respiratory virus first identified in 2001 (19). It belongs to the Pneumoviridae family and has a single-stranded, negative-sense RNA genome. Today it is recognized as one of the leading causes of hospitalization for respiratory tract infections (RTIs) among children <5 years of age (20, 21). CAMP expression has been shown to be downregulated in intestinal epithelial cells upon enteric bacterial infections (22–25) and in macrophages and dendritic cells upon Mtb infection (26–28). There have been few reports on how viral infections modulate CAMP expression. One study suggested that infection with respiratory syncytial virus (RSV), a respiratory virus closely related to hMPV, increased the transcriptional expression of both CYP27B1 and CAMP in human tracheobronchial epithelial (hTBE) cells (29). Another report showed that infection with influenza A virus led to reduced cCRAMP (a CAMP homolog in chinchilla) expression in chinchilla middle ear epithelial cells, while incubation with RSV or adenovirus only minimally affected cCRAMP level (30). A recent study showed that RSV infection led to increased mCRAMP (the murine homolog of CAMP) expression in mouse lungs (31). Although type I interferon has been suggested to suppress vitamin D-dependent CAMP response in human monocytes/macrophages (32), to our knowledge it is still unknown if viral infections modulate CAMP expression in these cells. This is particularly pertinent to human alveolar macrophages, which constantly patrol the microenvironment of the lung and act as a first line of defense against various types of respiratory pathogens, including viruses that are usual triggers of RTIs in humans. In addition, the mechanisms underlying pathogen-modulated CAMP expression are poorly understood.

In this study, we show for the first time that infection with hMPV strongly suppresses basal and vitamin-D induced CAMP expression in human macrophages. The suppression is likely mediated through downregulation of C/EBPα, a transcription factor critical for CAMP expression.

## Results

### hMPV Infection Suppresses CAMP Expression in Human Macrophages

**Figure 1:**
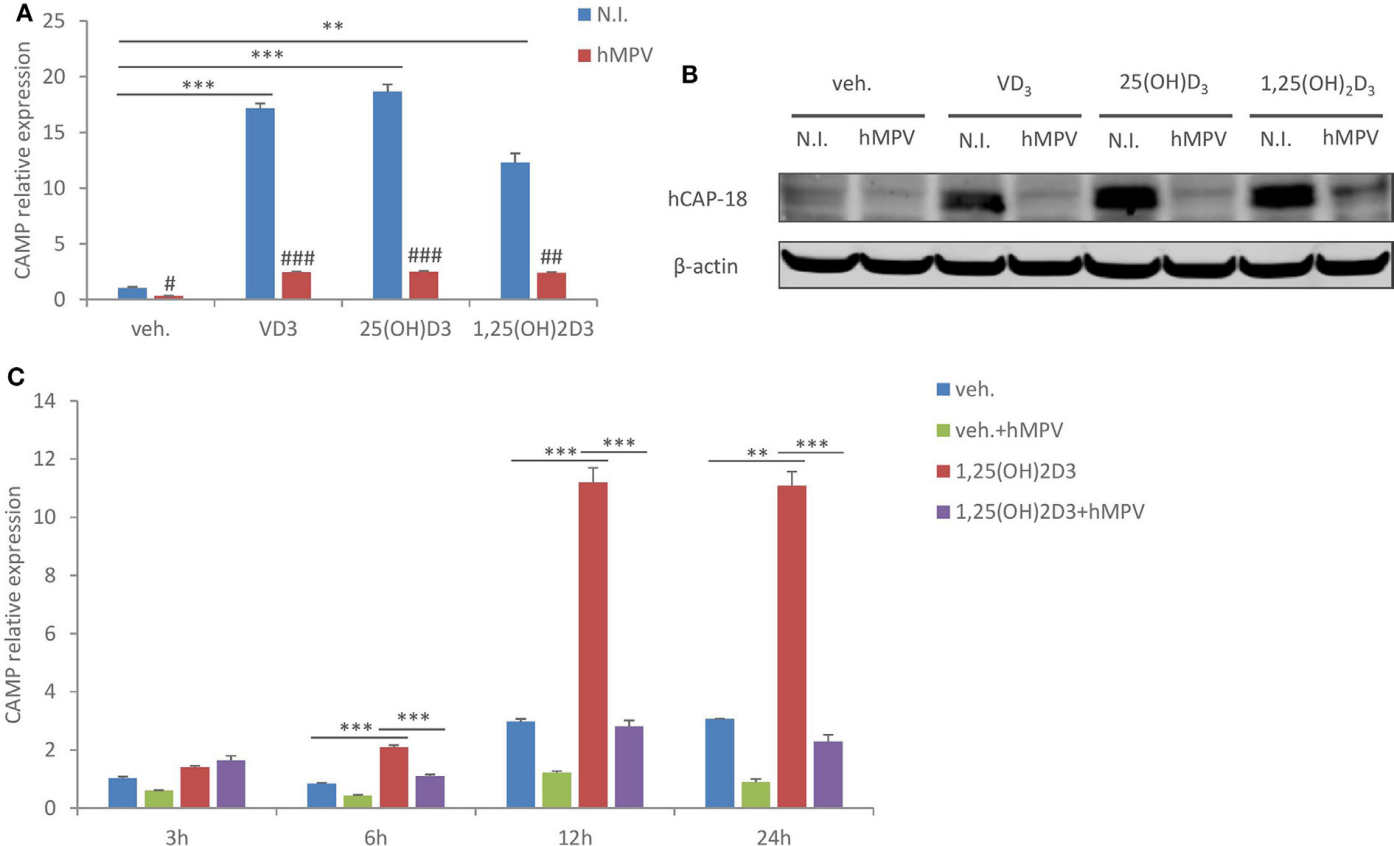
Human metapneumovirus (hMPV) infection suppresses cathelicidin antimicriobial peptide (CAMP) expression in human macrophages. **(A,B)** Monocyte-derived macrophages (MDMs) were treated with vehicle (veh.) or different forms of vitamin D [VD_3_, 25(OH)D_3_, and 1,25(OH)_2_D_3_], and concomitantly infected by hMPV or mock-infected by medium [non-infected (N.I.)] for 24 h. CAMP mRNA expression **(A)** and protein expression (hCAP-18) **(B)** were assessed by quantitative real-time PCR (qRT-PCR) and immunoblotting. **(C)** MDMs were treated with veh. or 1,25(OH)_2_D_3_ and infected by hMPV or mock infected for indicated hours. CAMP mRNA expression was assessed by qRT-PCR. Error bars represent SD for triplicates. **p < 0.01, ***p < 0.001; ^#^*p* < 0.05, ^##^*p* < 0.01, and ^###^*p* < 0.001 (compared to non-infected samples with the same veh. or vitamin D treatment). Data are representative of at least three independent experiments from different donors. Full-length blots are presented in Figure S1 in Supplementary Material.

To examine the effect of hMPV infection on CAMP expression in human macrophages, we infected human monocyte-derived macrophages (MDMs) with hMPV at MOI 1, in the presence or absence of 100 nM of VD_3_ (the precursor form of vitamin D), 25(OH)D_3_ (circulating vitamin D), or 1,25(OH)_2_D_3_ (active vitamin D). Cells were treated under serum-free conditions to rule out the potential confounding effects from serum vitamin D. As shown in Figures 1A,B, while the basal expression level of CAMP was low, all three forms of vitamin D potently induced mRNA expression of CAMP and protein expression of the precursor (hCAP-18). This is consistent with an earlier report showing that human macrophages possess the enzymatic machineries to convert both VD_3_ and 25(OH)D_3_ into the active metabolite 1,25(OH)_2_D_3_ (33). Our immunoblot did not reveal the mature peptide LL-37 (not shown). Interestingly, hMPV infection considerably repressed both the constitutive and vitamin D-induced CAMP expression (Figures 1A,B). A kinetic study further showed that vitamin D-induced CAMP expression and hMPV-mediated suppression which appeared early and became evident at 12 and 24 h (Figure 1C). These data demonstrate that hMPV infection strongly suppresses CAMP expression in human macrophages.

### hMPV-Mediated Suppression of CAMP Is Dependent on Viral Replication

**Figure 2:**
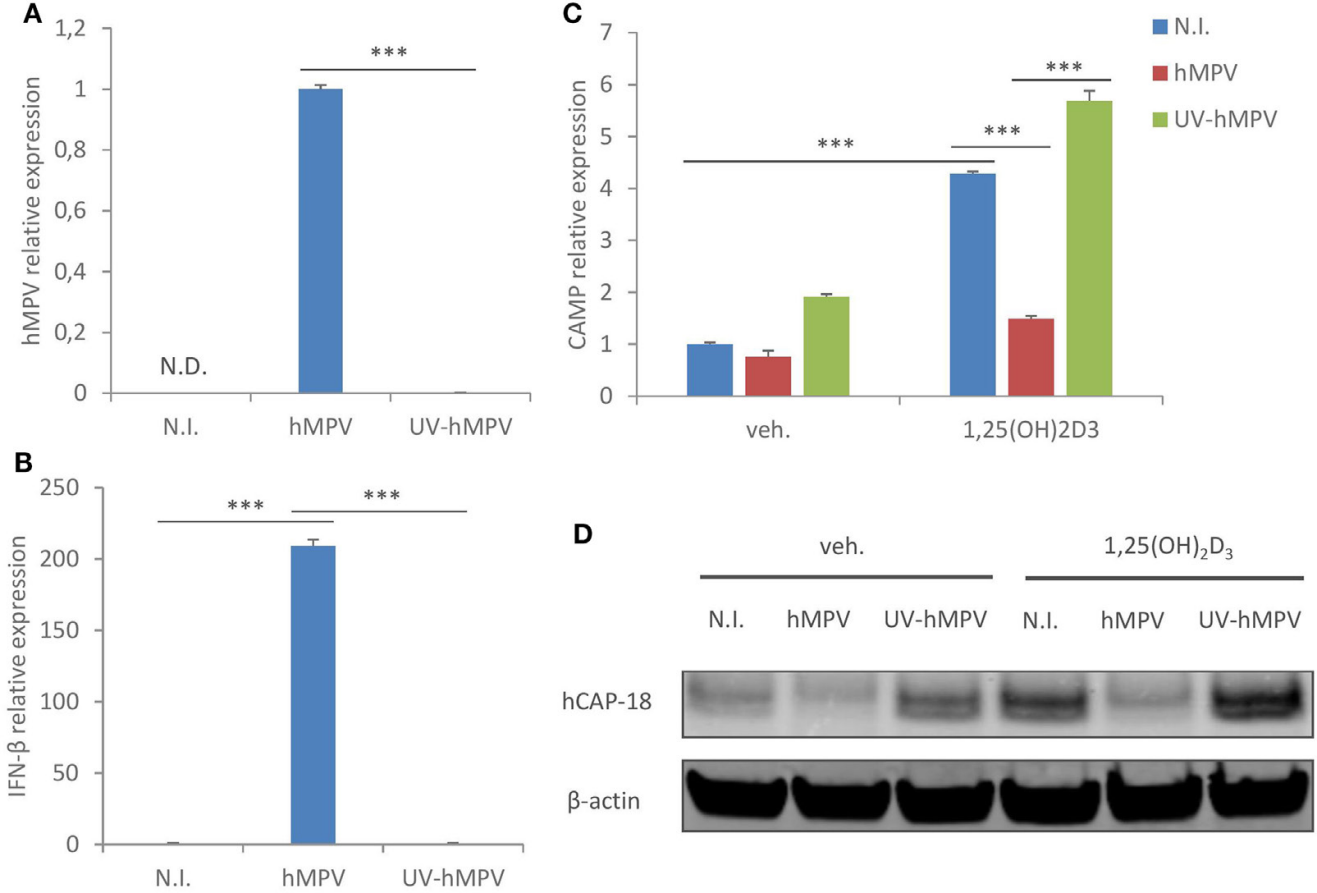
Human metapneumovirus (hMPV)-mediated suppression of cathelicidin antimicriobial peptide (CAMP) is dependent on viral replication. **(A,B)** Monocyte-derived macrophages (MDMs) were mock-infected (N.I.), infected with hMPV or inoculated with UV-irradiated hMPV (UV-hMPV) for 24 h. mRNA expression of hMPV N protein (hMPV) **(A)** or interferon-β (IFN-β) **(B)** was assessed by quantitative real-time PCR (qRT-PCR). **(C,D)** MDMs were mock-infected (N.I.), infected with hMPV or inoculated with UV-irradiated hMPV (UV-hMPV) in the presence or absence of 1,25(OH)_2_D_3_ for 24 h. CAMP mRNA expression **(C)** and protein expression (hCAP-18) **(D)** were assessed by qRT-PCR and immunoblotting. Error bars represent SD for triplicates. N.D., not detected. ***p < 0.001. Data are representative of at least three independent experiments from different donors. Full-length blots are presented in Figure S1 in Supplementary Material.

We next assessed if the suppressive effect of hMPV on CAMP expression was dependent on viral replication. MDMs were inoculated with wild-type hMPV or UV-irradiated hMPV (a routine method to block viral replication), and the expression of viral gene (hMPV N protein) and interferon-β was assessed by qRT-PCR. As shown in Figure 2A, hMPV gene expression was detected in MDMs infected with wild-type hMPV, while the expression was nearly abrogated in MDMs inoculated with UV-irradiated hMPV. Consistent with this result, UV-irradiated hMPV failed to induce interferon-β expression in MDMs (Figure 2B). UV-irradiated hMPV also lost the suppressive effect on CAMP expression both at mRNA and protein level (Figures 2C,D), suggesting that the suppressive effect is dependent on hMPV replication in human macrophages.

### hMPV Infection Does Not Downregulate Transcriptional Expression of CYP27B1 or VDR, but Inhibits VDR Protein Expression

**Figure 3:**
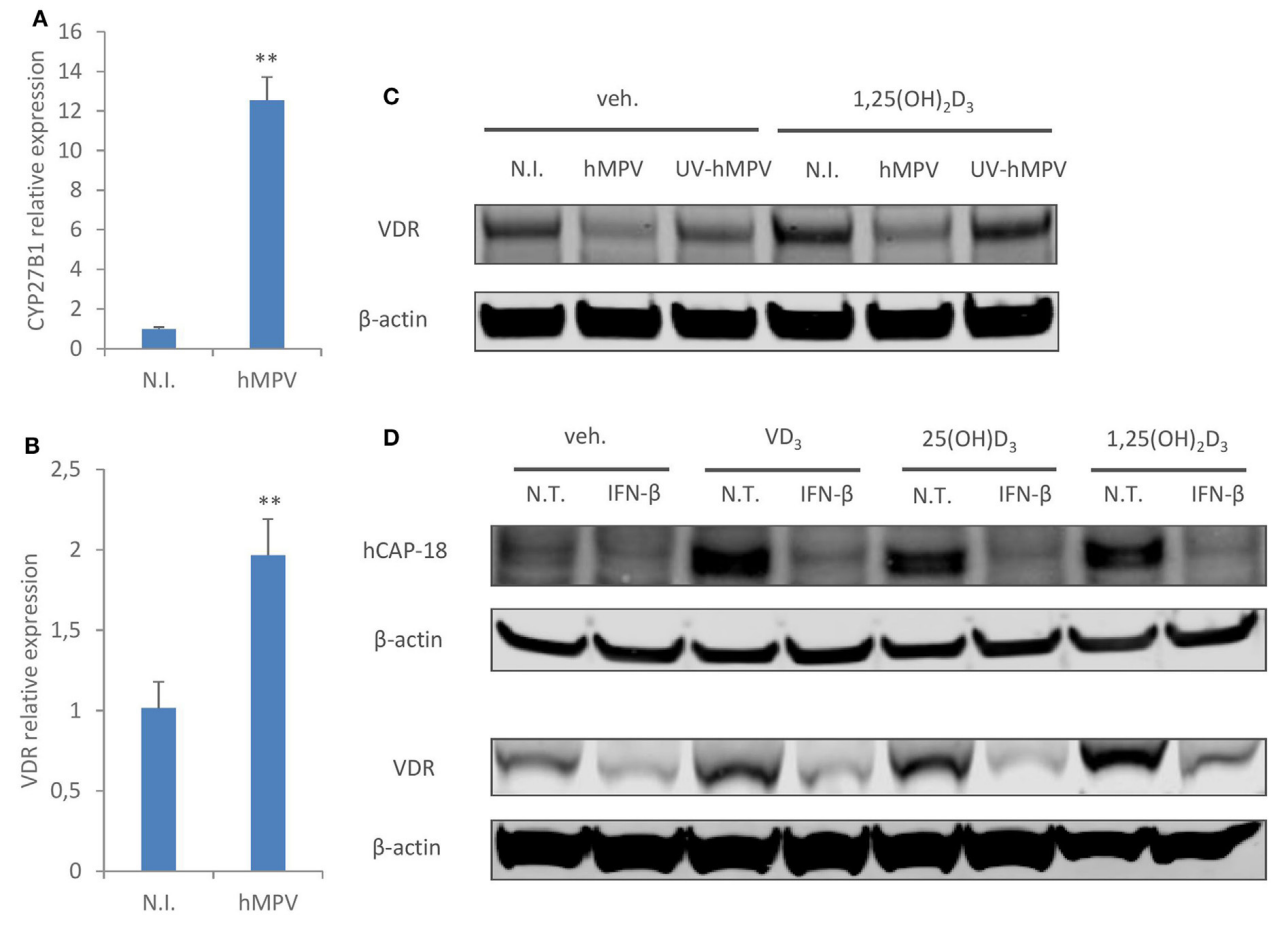
Human metapneumovirus (hMPV) infection does not downregulate transcriptional expression of CYP27B1 or vitamin D receptor (VDR), but inhibits VDR protein expression. **(A,B)** Monocyte-derived macrophages (MDMs) were mock-infected (N.I.) or infected with hMPV for 24 h. mRNA expression of CYP27B1 **(A)** or VDR **(B)** was assessed by quantitative real-time PCR. **(C)** MDMs were mock-infected (N.I.), infected with hMPV or inoculated with UV-inactivated hMPV (UV-hMPV) for 24 h in the presence or absence of 1,25(OH)_2_D_3_. VDR protein expression was assessed by immunoblotting. **(D)** MDMs were non-treated (N.T) or treated with recombinant interferon-β (IFN-β) for 24 h in the presence or absence of different forms of vitamin D. Cathelicidin antimicriobial peptide (hCAP-18) and VDR protein expression was assessed by immunoblotting. Error bars represent SD for triplicates. **p < 0.01. Data are representative of at least three independent experiments from different donors. Full-length blots are presented in Figure S1 in Supplementary Material.

Next, we sought to investigate the molecular mechanisms un-derlying hMPV-mediated suppression of CAMP. It has been previously shown that type I interferon suppresses type II interferon induced CAMP expression by inhibiting the transcriptional expression of CYP27B1 and VDR, thereby interfering with vitamin D-dependent induction of CAMP (32). Therefore, we went on to assess if hMPV infection downregulates the expression of CYP27B1 or VDR. Interestingly, hMPV-infected MDMs showed considerably enhanced expression of CYP27B1 and a modest upregulation of VDR at mRNA level (Figures 3A,B). The lack of positive correlation between CAMP and CYP27B1/VDR expression was not totally unexpected, as hMPV infection not only downregulated CAMP expression induced by inactive vitamin D (VD_3_ and 25(OH)D_3_), but also basal CAMP expression (VDR- and CYP27B1-independent) as well as 1,25(OH)_2_D_3_ induced CAMP expression (CYP27B1-independent) (Figures 1A,B). We did, however, observe reduced VDR protein expression upon hMPV infection, which was dependent on viral replication as UV-irradiated hMPV did not suppress VDR protein expression (Figure 3C). The decrease of VDR protein expression upon hMPV infection seemed to contradict the increase of VDR mRNA expression (Figure 3B). We reasoned that reduced VDR protein expression might result from interferon-mediated inhibition of protein synthesis (34), as MDMs treated with recombinant interferon-β also showed decreased protein expression of VDR as well as CAMP (Figure 3D). VDR may be particularly sensitive to interferon-mediated inhibition of protein synthesis, as it has been shown earlier that VDR protein has a high turnover rate and is rapidly degraded by the ubiquitin/proteasome system (35). Altogether these data suggest that hMPV-mediated suppression of CAMP cannot be explained by transcriptional modulation of CYP27B1 or VDR, although we cannot rule out the possibility that hMPV-mediated inhibition of VDR protein expression may contribute to impaired induction of CAMP by vitamin D.

### hMPV-Mediated Suppression of CAMP Is Largely Interferon-Independent

**Figure 4:**
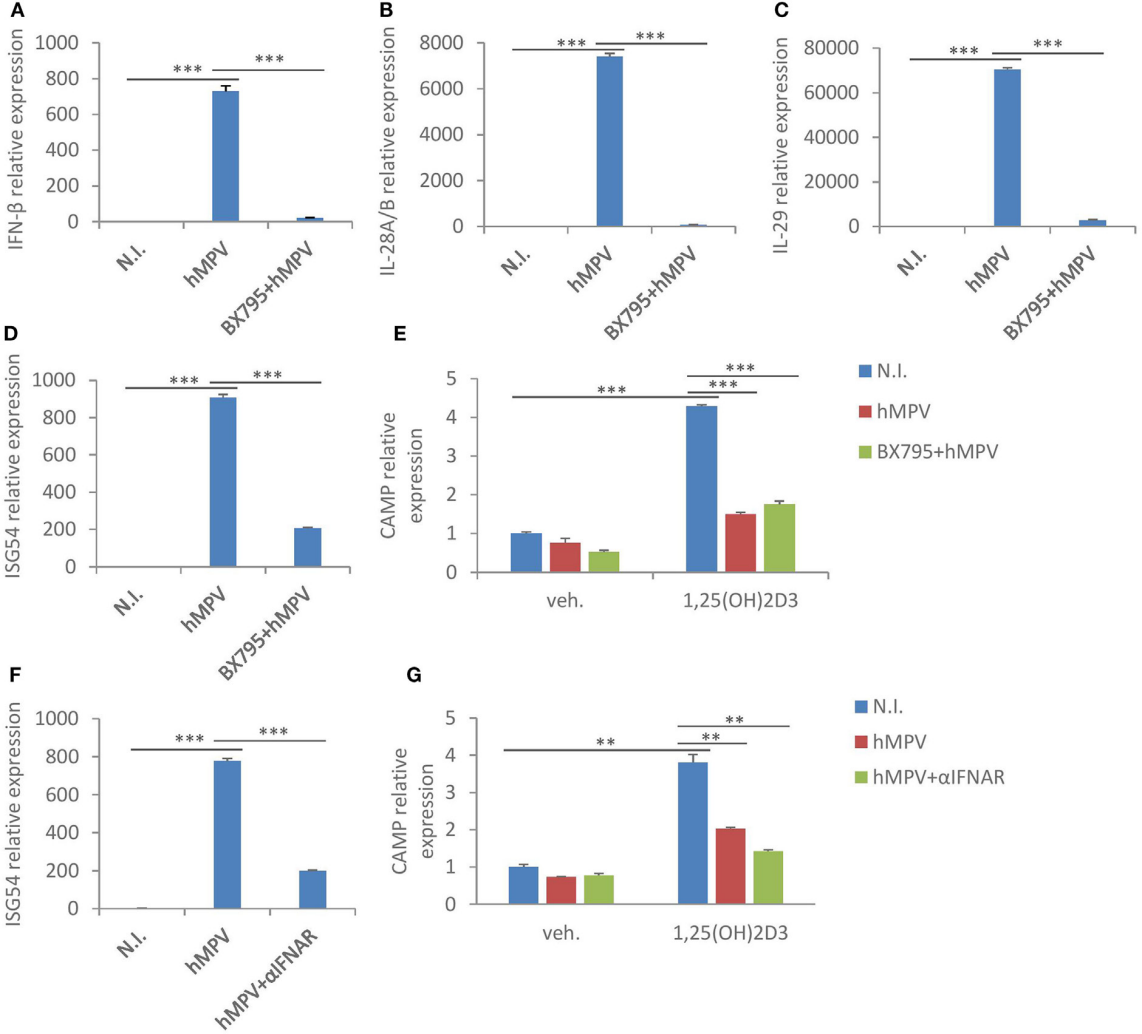
Human metapneumovirus (hMPV)-mediated suppression of cathelicidin antimicriobial peptide (CAMP) is largely interferon-independent. **(A–D)** Monocyte-derived macrophages (MDMs) were mock-infected (N.I.), infected with hMPV (hMPV) or pretreated with BX795 before hMPV infection (BX795 + hMPV) for 24 h. mRNA expression of IFN-β **(A)**, IL-28A/B **(B)**, IL-29 **(C)**, and ISG54 **(D)** was assessed by quantitative real-time PCR (qRT-PCR). **(E)** Same set-up for hMPV infection and BX795 pretreatment as **(A–D)**, concomitant with or without 1,25(OH)_2_D_3_ treatment. CAMP mRNA expression was assessed by qRT-PCR. **(F)** MDMs were mock-infected (N.I.), infected with hMPV (hMPV) or pretreated with IFNAR2 neutralizing antibody (αIFNAR) before hMPV infection (hMPV + αIFNAR) for 24 h. mRNA expression of ISG54 was assessed by qRT-PCR. **(G)** Same set-up for hMPV infection and αIFNAR pretreatment as **(F)**, concomitant with or without 1,25(OH)_2_D_3_ treatment. CAMP mRNA expression was assessed by qRT-PCR. Error bars represent SD for triplicates. **p < 0.01, ***p < 0.001. Data are representative of at least three independent experiments from different donors.

Macrophages are an important source of interferons following viral infections [Figure 2B (36)]. Since we found that interferon-β treatment had a similar suppressive effect on both CAMP and VDR protein expression (Figure 3D), we asked if hMPV-mediated suppression of CAMP was merely modulated by interferons. To evaluate the role of interferons, we used two distinct approaches to block interferon-signaling pathways. First, we pretreated MDMs with BX795, which potently blocks IRF3 activation and interferon production through inhibition of the IKK-related kinases TANK-binding kinase 1 and IKKε (37). Pretreatment with BX795 effectively blocked the induction of interferon β (Figure 4A), type III interferons—IL28a/b and IL29 (Figures 4B,C), and interferon inducible gene ISG54 (Figure 4D). Surprisingly, the suppressive effect of hMVP on CAMP was largely unaffected upon BX795 pretreatment (Figure 4E), suggesting little involvement of type I or type III interferons. We next used a neutralizing antibody for the receptor of type I interferons (αIFNAR) to block interferon-signaling pathways. Pre-treatment with αIFNAR also potently inhibited interferon signaling, as evidenced by considerably red-uced expression of ISG54 upon hMPV infection (Figure 4F), suggesting that type I interferons were the major contributors to the interferon-mediated response. However, blockade of IFNAR signaling did not reverse the suppressive effect of hMPV on CAMP (Figure 4G). Taken together these data suggest that interferons are not the main contributors to hMPV-mediated inhibition of CAMP.

### hMPV Infection Inhibits C/EBPα Expression, Which Is Critical for CAMP Expression in Human Macrophages

**Figure 5:**
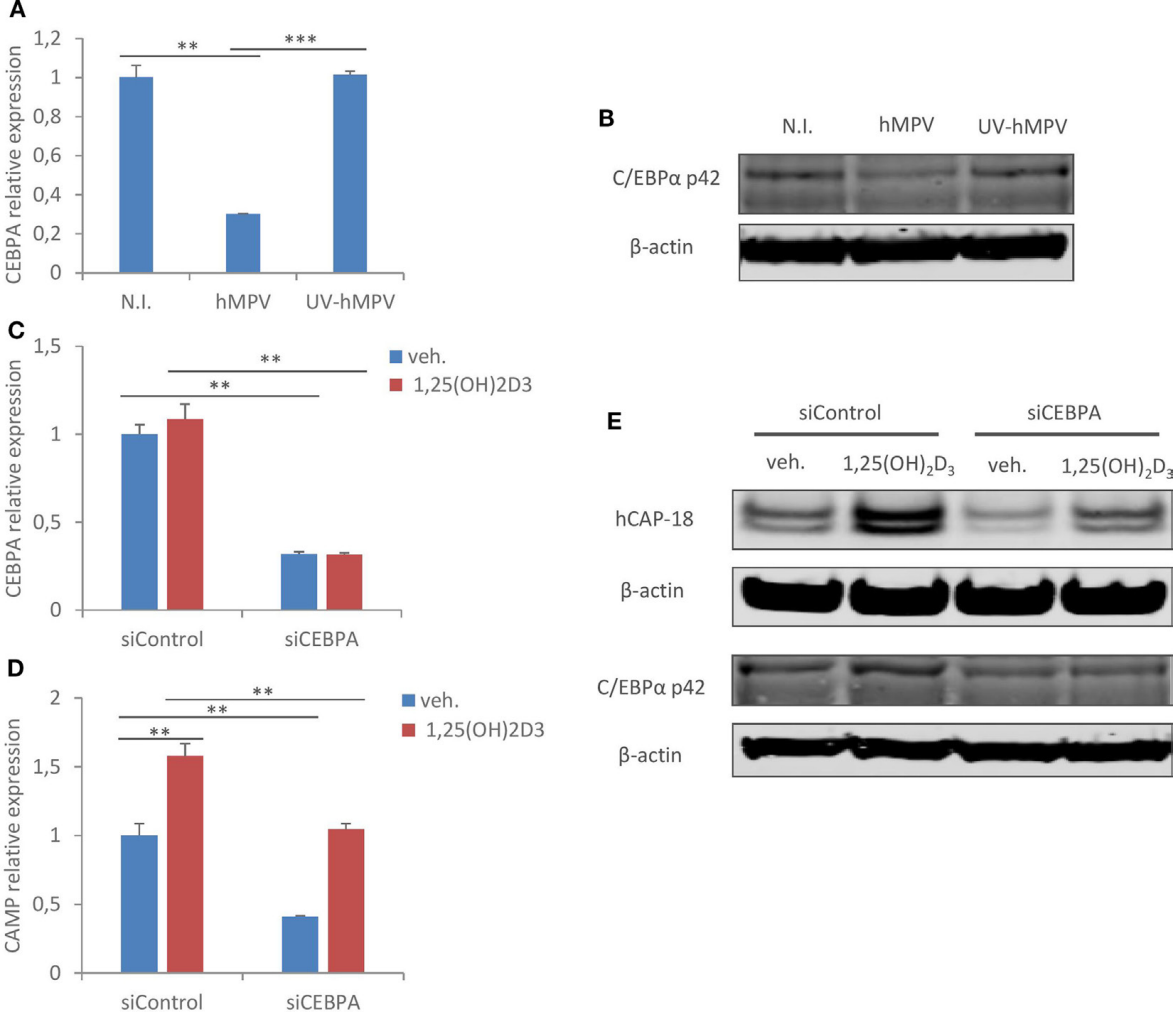
Human metapneumovirus (hMPV) infection inhibits C/EBPα expression, which is critical for cathelicidin antimicriobial peptide (CAMP) expression in human macrophages. **(A,B)** Monocyte-derived macrophages (MDMs) were mock-infected (N.I.), infected with hMPV (hMPV) or inoculated with UV-inactivated hMPV (UV-hMPV) for 24 h. C/EBPα mRNA (CEBPA) expression **(A)** and protein expression (p42) **(B)** was assessed by quantitative real-time PCR (qRT-PCR) and immunoblotting. **(C–E)** MDMs were transfected with siRNA targeting C/EBPα (siCEBPA) or scrambled control siRNA (siControl) for 72 h before vehicle or 1,25(OH)_2_D_3_ treatment. mRNA expression of C/EBPα (CEBPA) **(C)** and CAMP **(D)** was assessed by qRT-PCR. C/EBPα (p42) and CAMP (hCAP-18) protein expression was assessed by immunoblotting **(E)**. Error bars represent SD for triplicates. **p < 0.01, ***p < 0.001. Data are representative of at least three independent experiments from different donors. Full-length blots are presented in Figure S1 in Supplementary Material.

We went on to seek alternative explanations to hMPV-mediated suppression of CAMP in human macrophages. The transcription factor C/EBPα has been shown to be a potent enhancer of CAMP transcription (38). Therefore, we evaluated if hMPV infection alters the expression of C/EBPα in human macrophages. As shown in Figure 5A, infection with wild type, but not UV-irradiated hMPV, significantly reduced mRNA expression of C/EBPα in MDMs. In addition, immunoblot showed reduced protein expression of C/EBPα p42, the isoform that possesses transactivation potential (39), upon wild-type hMPV infection (Figure 5B). These data suggest that hMPV infection may suppress CAMP expression through downregulation of C/EBPα. To validate the importance of C/EBPα to CAMP expression in human macrophages, we used siRNA to knockdown C/EBPα. Silencing efficiency was validated by measuring both mRNA and protein (p42) expression of C/EBPα in siRNA transfected MDMs (Figures 5C,E). Importantly, knockdown of C/EBPα resulted in reduced expression of CAMP both at mRNA and protein level in the presence or absence of 1,25(OH)_2_D_3_ (Figures 5D,E). These data indicate that C/EBPα is indeed an important transcription factor regulating CAMP expression, and that the suppressive effect of hMPV on CAMP expression can be explained, at least in part, by hMPV-mediated downregulation of C/EBPα.

### TLR1/2 Ligand Treatment Reproduces the Same Suppressive Effect on C/EBPα and CAMP in Human Macrophages

**Figure 6:**
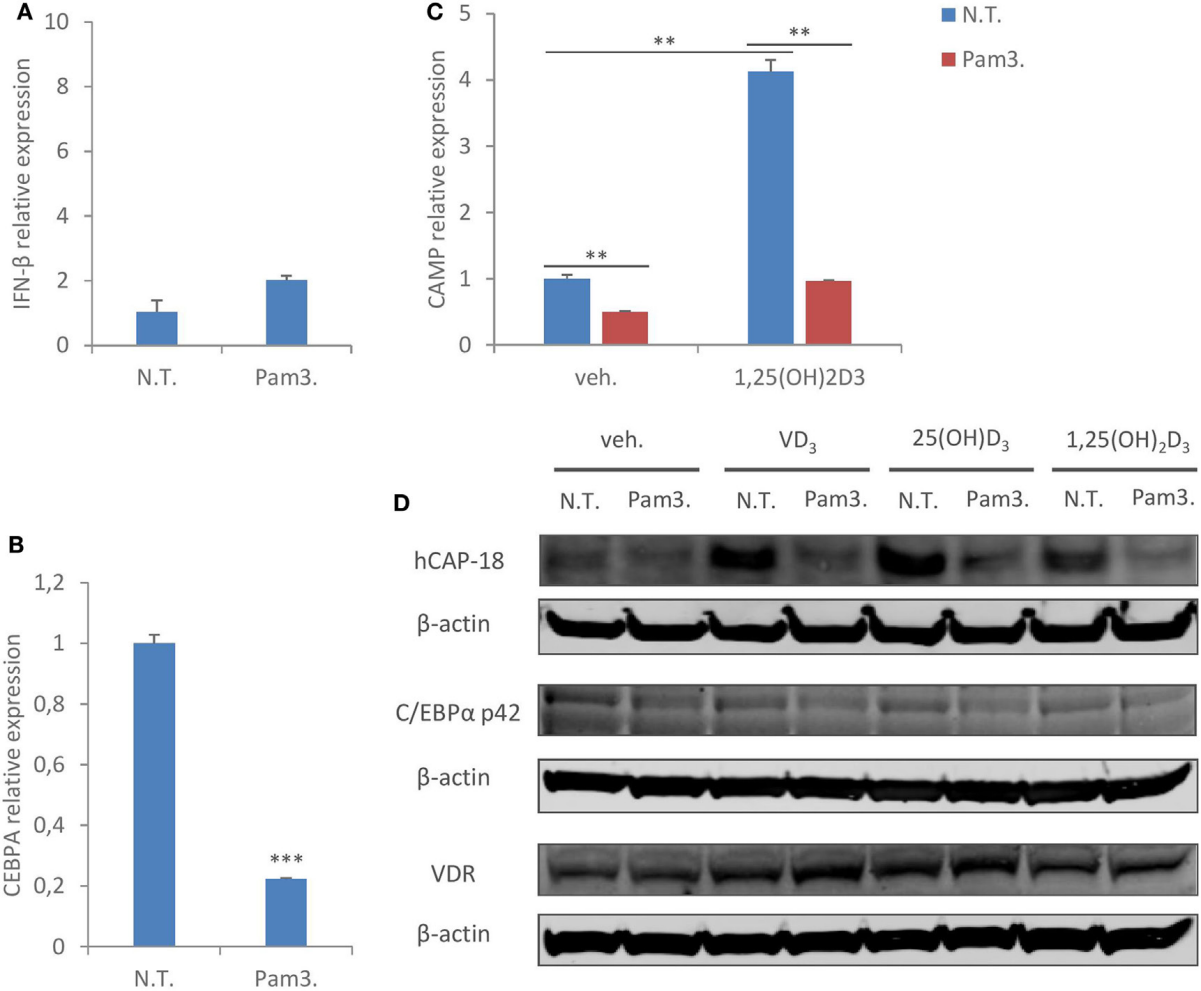
TLR1/2 ligand treatment reproduces the same suppressive effect on C/EBPα and cathelicidin antimicriobial peptide (CAMP) in human macrophages. **(A,B)** Monocyte-derived macrophages (MDMs) were non-treated (N.T.) or treated with TLR1/2 ligand Pam3CSK4 (Pam3.) for 24 h. IFN-β **(A)** and C/EBPα (CEBPA) **(B)** mRNA expression was assessed by quantitative real-time PCR (qRT-PCR). **(C)** MDMs were non-treated (N.T.) or treated with Pam3CSK4 for 24 h in the presence or absence of 1,25(OH)_2_D_3_. mRNA expression of CAMP was assessed by qRT-PCR. **(D)** MDMs were non-treated (N.T.) or treated with Pam3CSK4 for 24 h in the presence or absence of different forms of vitamin D. CAMP (hCAP-18), C/EBPα (p42), and vitamin D receptor (VDR) protein expression was assessed by immunoblotting. Error bars represent SD for triplicates. **p < 0.01, ***p < 0.001. Data are representative of at least three independent experiments from different donors. Full-length blots are presented in Figure S1 in Supplementary Material.

It has been shown earlier that inflammatory stimuli such as toll-like receptor (TLR) ligands downregulate C/EBPα expression (40–42). As our knockdown study showed that C/EBPα is critical to CAMP expression, we next assessed if TLR activation inhibits C/EBPα expression, and concomitantly suppresses CAMP expression in human macrophages. TLR1/2 ligand Pam3CSK4 was chosen because it hardly induced interferon-β expression in MDMs (Figure 6A), excluding any potential interferon-mediated effects. As expected, Pam3CSK4 treatment suppressed C/EBPα mRNA expression (Figure 6B). Importantly, Pam3CSK4 treatment also inhibited CAMP mRNA expression in the presence or absence of 1,25(OH)_2_D_3_ (Figure 6C). In line with this, Immunoblot analysis showed reduced protein expression of CAMP and C/EBPα (p42) with Pam3CSK4 treatment (Figure 6D). In contrast to what was observed during hMPV infection or interferon-β treatment (Figures 3C,D), Pam3CSK4 treatment did not inhibit VDR protein expression (Figure 6D). Taken together these data suggest that inflammatory stimuli other than viruses may also modulate CAMP expression through C/EBPα downregulation in human macrophages.

## Discussion

In this study, we show that hMPV infection suppresses CAMP expression in human macrophages and provide evidence for the underlying mechanisms regulating this. Our investigations reveal several interesting findings. First, CAMP can be induced by all three forms of vitamin D, including the pre-hormone VD_3_. To our knowledge this is the first report showing that VD_3_, which is naturally synthesized in the skin or taken up as dietary supplement, is capable of inducing CAMP in human macrophages at a concentration that is physiologically relevant (100 nM) (43). Though perhaps less relevant to alveolar macrophages, this observation may have strong implications to resident macrophages in the skin or in the gastrointestinal system, where the cells are likely to be exposed to high concentrations of VD_3_. It is worth further investigation if the resident macrophages at the epidermal or gastrointestinal barriers produce more CAMP for local defense, owing to higher local VD_3_ levels.

Second, the expression of CAMP has been previously suggested to be strongly associated with the expression and function of CYP27B1 and VDR in human monocytes/macrophages (17, 32, 44). It was also reported earlier that RSV infection increased transcriptional expression of both CYP27B1 and CAMP in hTBE cells (29). Nevertheless, we did not observe a similar correlation between CYP27B1/VDR and CAMP expression in hMPV-infected human macrophages, at least not at the transcriptional level. In line with our data, a recent report also showed that in dendritic cells, TLR1/2 ligands upregulated CYP27B1 expression and 1,25(OH)_2_D_3_ production, yet downregulated CAMP expression (26). This and our work indicate that there are factors other than CYP27B1 or VDR affecting CAMP expression. C/EBPα is an attractive candidate as it has been previously shown to promote CAMP expression both alone and in synergy with VDR in human lung epithelial cells (38). Here, we show that hMPV infection strongly represses C/EBPα expression, and importantly our knockdown study confirms that C/EBPα is indeed important in regulating CAMP expression in human macrophages. These observations provide a mechanistic explanation to hMPV-mediated downregulation of CAMP.

Last but not least, although it is yet unclear how hMPV modulates the expression of C/EBPα in human macrophages, it is interesting to note that such downregulation is also observed when macrophages are activated by other inflammatory stimuli, such as TLR ligands [this study and Ref. (40–42)]. It was originally proposed that bacteria-mediated downregulation of antimicrobial effectors such as CAMP may represent an escape mechanism, which gives pathogens a survival advantage (22–25). Our study suggests another possibility that, at least in macrophages, this downregulation of CAMP could be a general effect downstream C/EBPα in response to different types of inflammatory stimuli. Macrophages play essential roles in tissue homeostasis and host defense, and their manifold functions need to be carefully programmed to react to different environmental cues. It is tempting to speculate that upon pathogenic stimulations, macrophages temporarily downregulate homeostatic effector molecules, such as CAMP, and prioritize other effector mechanisms such as production of interferons or recruitment of other immune cells through chemokines and cytokines. C/EBPα may be one of the master transcriptional regulators fine-tuning macrophage activities. The fact that blockade of interferon signaling hardly reverses hMPV’s suppressive effect on CAMP also suggests that the modulation is not a secondary response, but likely an integrated event of the “reprogramming” when macrophages sense pathogenic insults (in this case intracellular viral replication).

In summary, we show here that hMPV infection inhibits CAMP expression in human macrophages, possibly through modulation of C/EBPα. To our knowledge this is the first study demonstrating that in immune cells infection with a highly relevant human respiratory virus interferes with the antimicrobial peptide defense mechanism. Our study also provides mechanistic insights into the modulation of CAMP upon hMPV infection, which may apply to other types of infections. This virus-mediated downregulation of CAMP could have multiple consequences, given the multiple roles CAMP plays in the immune system. Further studies are needed to investigate if this altered antimicrobial peptide response following viral infections may affect host defense (e.g., against a secondary infection) under physiological conditions.

## Materials and Methods

### Reagents

Vitamin D and its metabolites [VD_3_, 25(OH)D_3_, and 1,25(OH)D_3_] were purchased from Tocris Bioscience and used at a working concentration of 100 nM. Pam3CSK4 was purchased from Invivogen and used at a working concentration of 500 ng/mL. BX795 was purchased from Axon Medchem and used at a working concentration of 2 µM (30 min pretreatment prior to hMPV infection). Type I interferon receptor (IFNAR2) neutralizing antibody (#21385-1) was purchased from PBL Assay Science and used at a working concentration of 10 µg/mL (30 min pretreatment prior to hMPV infection).

### Virus Propagation and Titration

Recombinant hMPV RecNL/1/00 (A1) was kindly provided by B. van den Hoogen (Erasmus MC, Rotterdam). LLC-MK2 monolayers were inoculated with virus at MOI 0.01 in OptiMEM (Thermo Fisher) containing 2% FBS, 20 µg/mL gentamicin, and 0.68 mM glutamine. Virus was harvested after 7–9 days, purified on a 20% sucrose cushion, and resuspended in OptiMEM (serum free). Purified virus was serially diluted (log10) on monolayers of LLC-MK2 cells in 96-well plates. Cells were washed after 4 days, stained with LIGHT DIAGNOSTICS™ hMPV direct fluorescence assay (Merck Millipore) and foci forming units were determined by manual counting.

### Cell Culture and *In Vitro* Infection

LLC-MK2 cells were cultivated in supplemented OptiMEM (5% FBS, 0.68 mM l-glutamine, and 20 µg/mL gentamicin). Peripheral blood mononuclear cells (PBMCs) were isolated from fresh buffy coats of healthy donors using gradient centrifugation with Lymphoprep™ (Axis-Shield). Buffy coats were supplied by the blood bank at St. Olavs Hospital in Trondheim, Norway, and their use in research has been approved by the Regional Committee for Medical and Health Research Ethics (REK), and by the donors themselves. Cells were washed with PBS and seeded in RPMI 1640 medium (supplemented with 0.34 mM l-glutamine and 10 µg/mL gentamicin). After 2 h non-adherent cells were removed by washing with RPMI 1640 medium. Monocytes were cultivated in RPMI 1640 medium supplemented with 10% human serum (heat inactivated, obtained from blood bank of St. Olavs Hospital, Trondheim), 0.34 mM l-glutamine, 10 µg/mL gentamicin, and 10 ng/mL M-CSF (Biolegend) for macrophage differentiation. Medium was changed every 3 days. Macrophages differentiated for 9–12 days were used in this study. On the day of treatment, medium was switched to serum-free OptiMEM. Cells were inoculated with wild-type or UV-irradiated hMPV at MOI 1 for 24 h unless indicated otherwise.

### Quantitative Real-Time PCR (qRT-PCR)

RNA was isolated with the RNeasy mini kit (Qiagen) following the manufacturer’s protocol. cDNA was synthesized from isolated RNA using the qScript kit (Quanta) following the manufacturer’s protocol. qRT-PCR was performed using Perfecta SYBR Green reaction mix (Quanta) and a StepOnePlus instrument (Life Technologies) with the temperature profile at 95°C for 20 s, 40 cycles at 95°C for 3 s, and at 60°C for 30 s. Fold change in gene expression was calculated using the ΔΔCt-method normalized against GAPDH. Primer sequences are listed in Table S1 in Supplementary Material.

### RNA Interference

siRNAs were purchased from Qiagen (AllStars control siRNA) and Ambion (CEBPA), respectively. siRNA duplexes were reverse transfected into cells using Lipofectamine RNAiMAX (Thermo Fisher Scientific) transfection reagent according to the manufacturer’s instructions. Transfected cells were allowed to grow for another 72 h before infection or treatment.

### Western Blot

Cells were washed once in PBS and lysed in lysis buffer (50 mM Tris–HCl, 150 mM NaCl, 10% glycerol, 0.5% Triton X-100, and 2 mM EDTA) containing phosphatase and protease inhibitors (100 mM sodium fluoride, 1 mM sodium orthovanadate, 40 mM β-glycerophosphate, 10 µg/mL leupeptin, 1 µM pepstatin A, and 1 mM phenylmethylsulfonyl fluoride). Protein extracts were separated by NuPAGE^®^ Bis-Tris gels (Thermo Fisher Scientific) and dry blotting was performed using iBlot^®^ Gel Transfer stacks Nitrocellulose Mini kit and iBlot^®^ machine (Invitrogen). Primary human antibodies for hCAP-18 (#650302) and C/EBPα (#662102) were purchased from Biolegend. Household β-actin antibody (A1978) was purchased from SIGMA-ALDRICH and used as a loading control. Secondary antibodies (IRDye^®^ 800CW Goat anti-Mouse, IRDye^®^ 680RD Goat anti-Mouse) were purchased from LI-COR Biosciences. LICOR Odyssey imager was used as the scanning system.

### Statistics

Results are expressed as mean + SD (*n* = 3). A two-sided *P*-value <0.05 as determined by Student’s *t*-test was considered significant. All data are representative for at least three independent experiments with PBMCs from different donors.

## Data Availability

Data supporting the conclusions of this manuscript will be made available by the authors, without undue reservation, to any qualified researcher.

## Ethics Statement

Buffy coats used in this study were supplied by the blood bank at St. Olavs Hospital in Trondheim, Norway, and their use in research has been approved by the Regional Committee for Medical and Health Research Ethics (REK), and by the donors themselves.

## Author Contributions

YL and IJ conceived the experiments. YL, SØ, and IJ conducted the experiments and analyzed the results. All authors reviewed and approved the manuscript.

## Conflict of Interest Statement

The authors declare that the research was conducted in the absence of any commercial or financial relationships that could be construed as a potential conflict of interest. The reviewer AN and handling Editor declared their shared affiliation.
