# Supplementary material for: Human Metapneumovirus Infection Inhibits Cathelicidin Antimicrobial Peptide Expression in Human Macrophages
Source: Front Immunol. 2018 May 4;9:902. doi: 10.3389/fimmu.2018.00902 (PMC5946005; doi:10.3389/fimmu.2018.00902)
Supplement: Supplementary file 1 [file image_1.PDF]

Supplementary Figure 1: full-length blots

Figure 1B

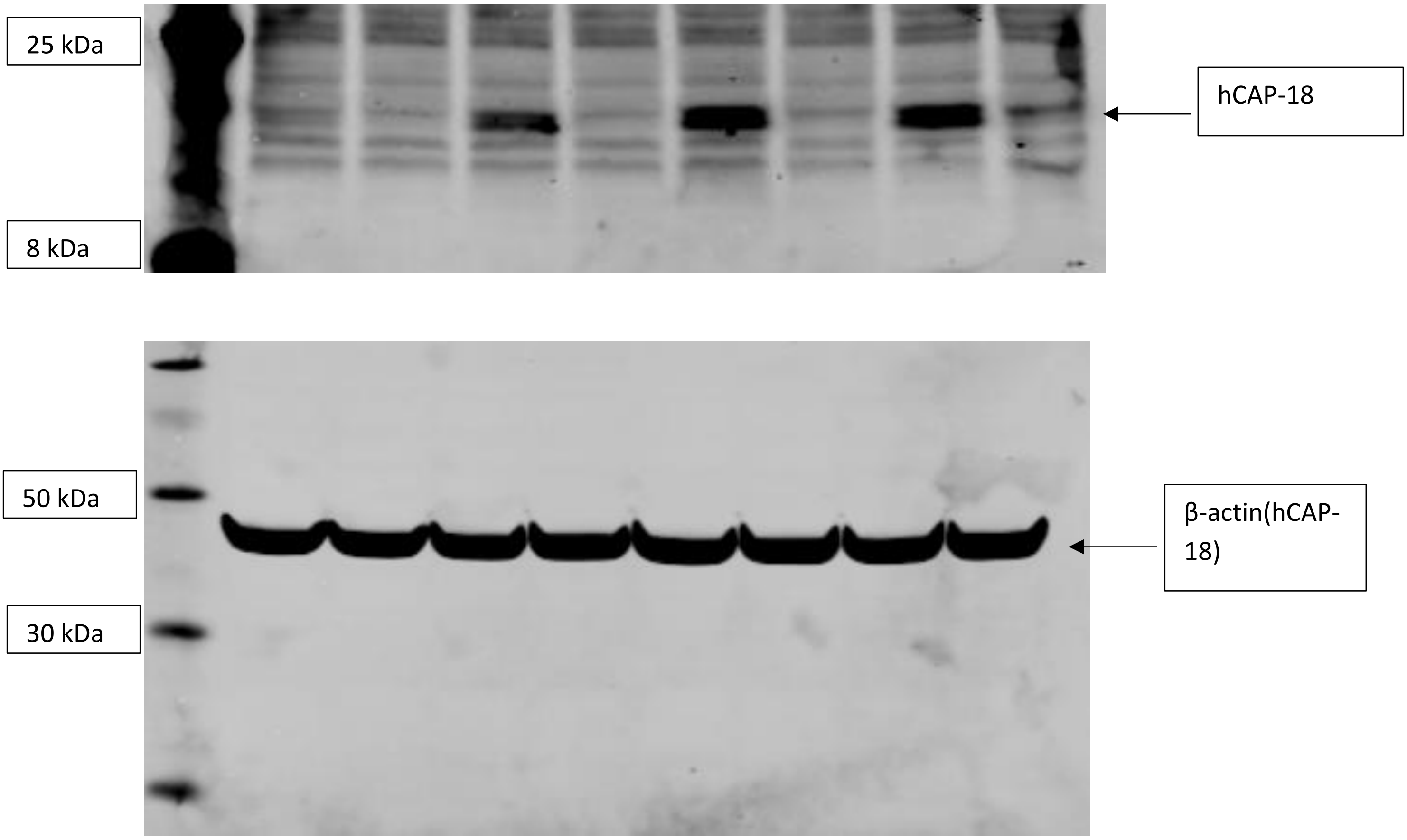

Figure 2D

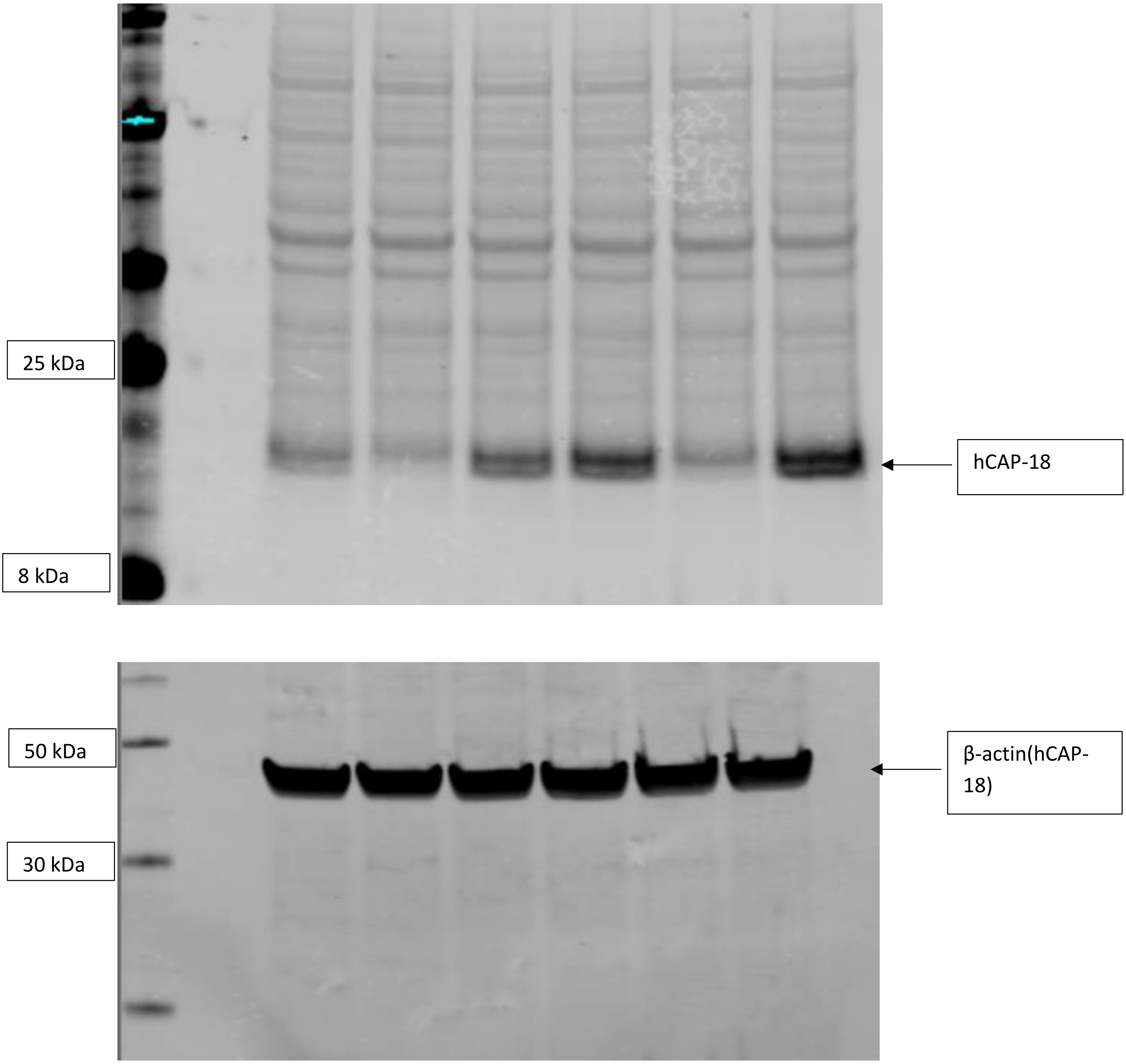

Figure 3C

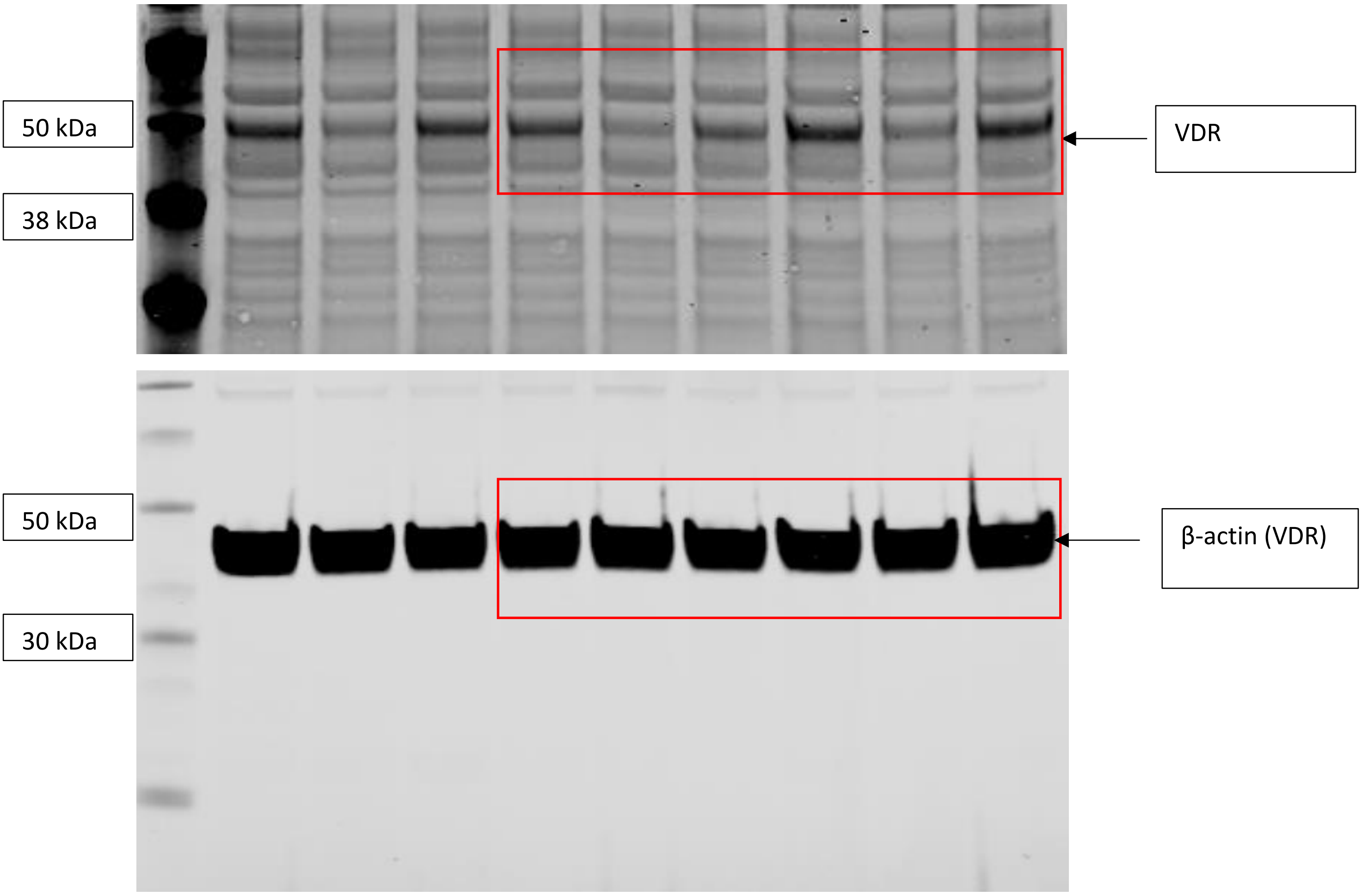

Figure 3D

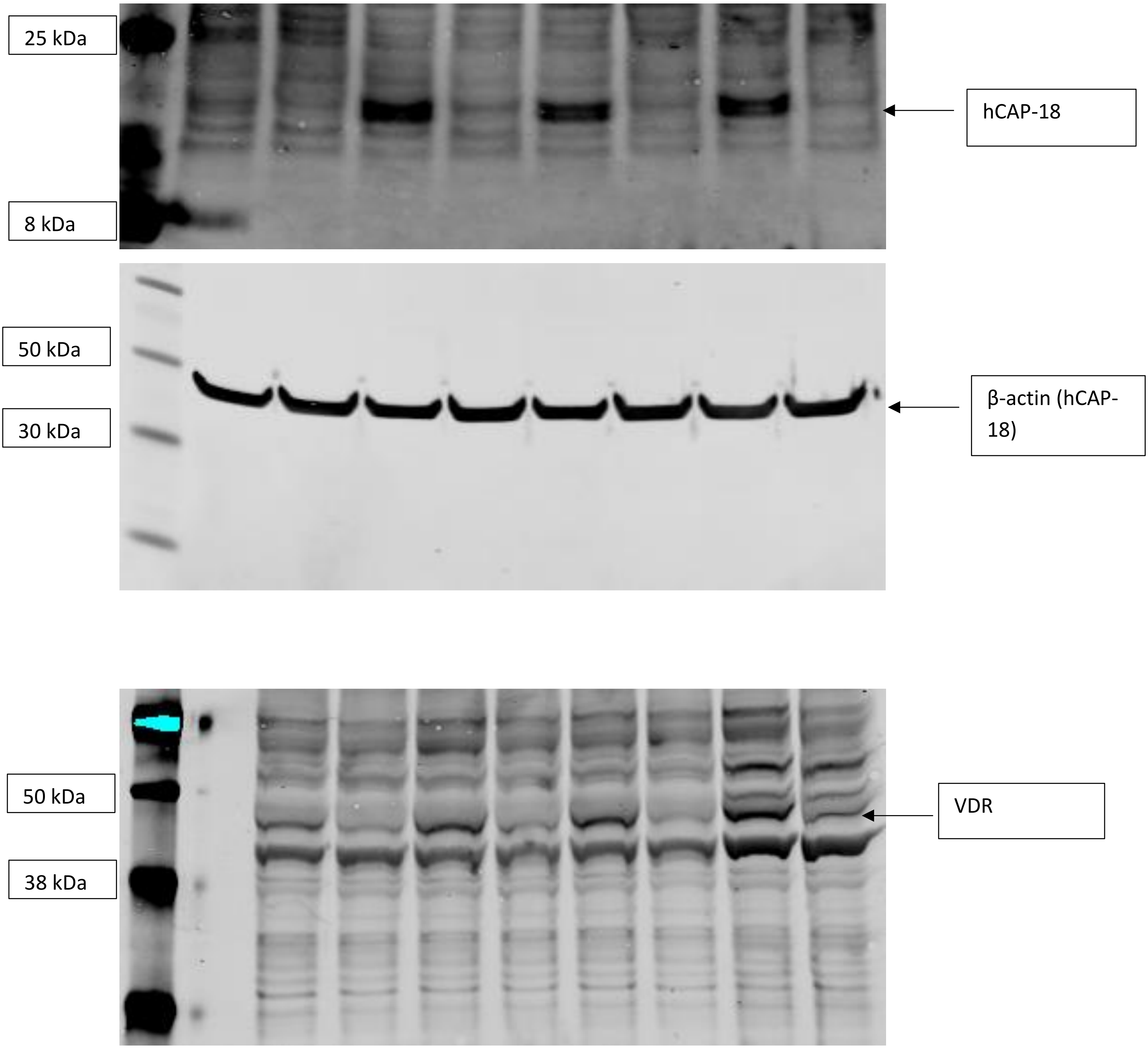
25 kDa
8 kDa
hCAP-18
50 kDa
30 kDa
β-actin (hCAP-18)
50 kDa
38 kDa
VDR

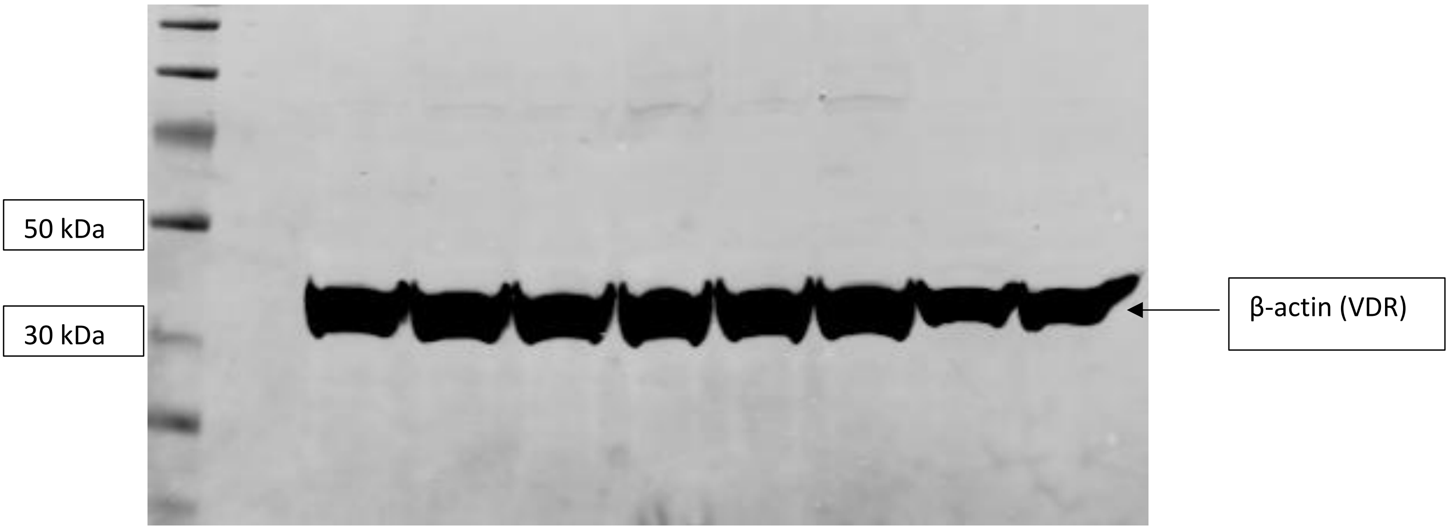
50 kDa
30 kDa
β-actin (VDR)

Figure 5B, E

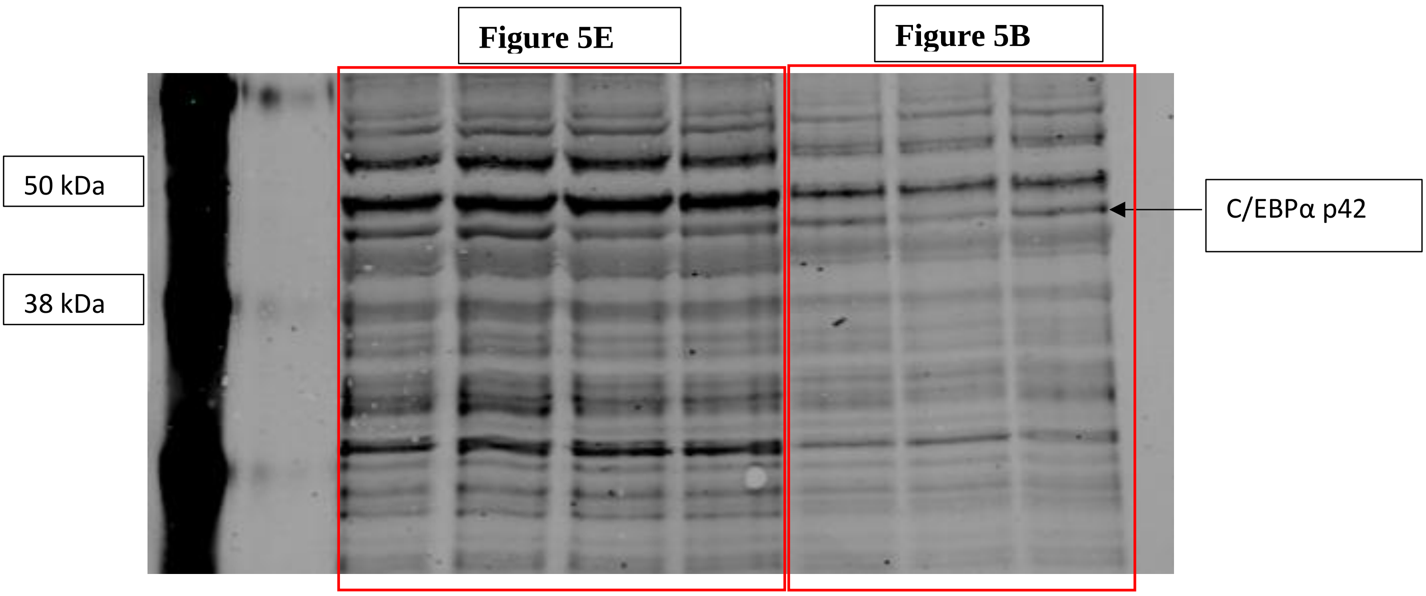
Figure 5E
Figure 5B
50 kDa
38 kDa
C/EBPα p42

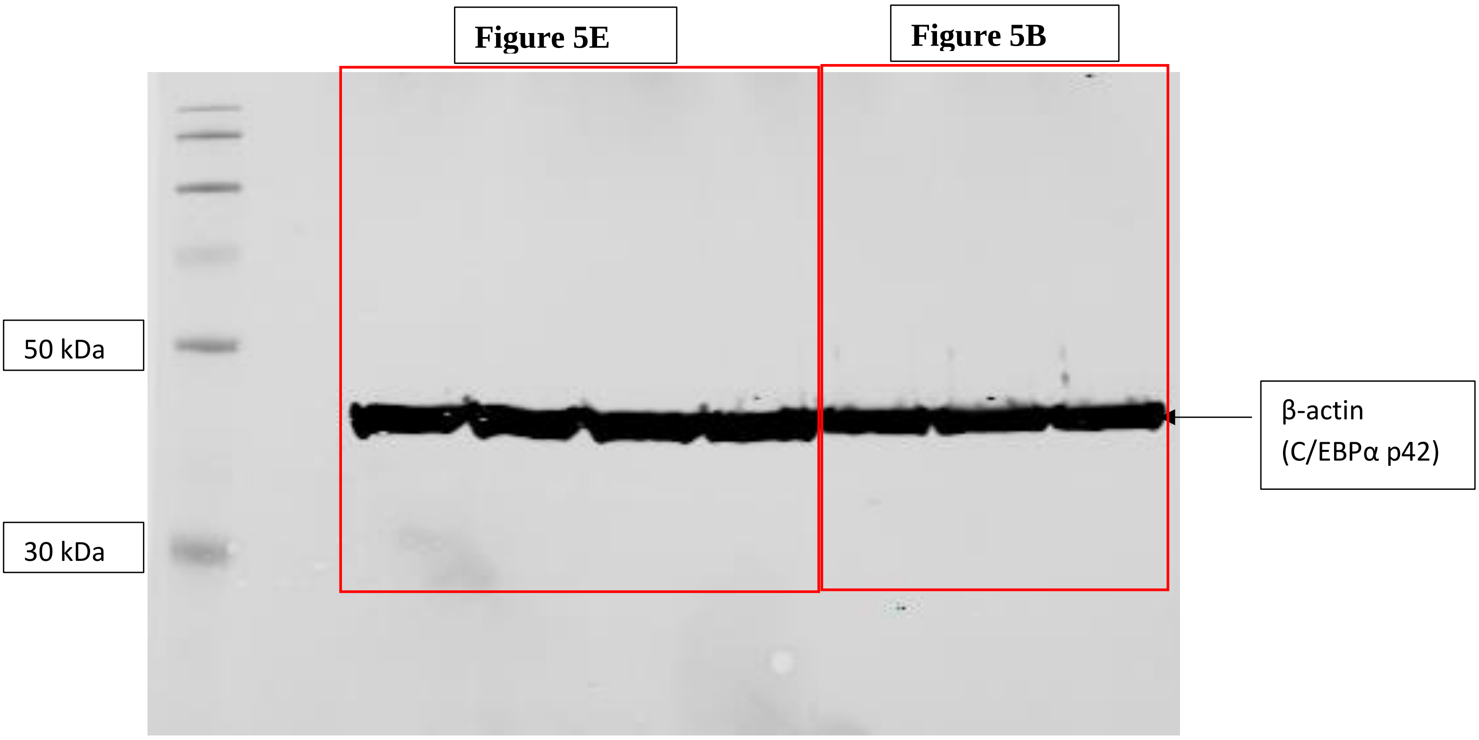
Figure 5E
Figure 5B
50 kDa
30 kDa
β-actin
(C/EBPα p42)

Figure 5E

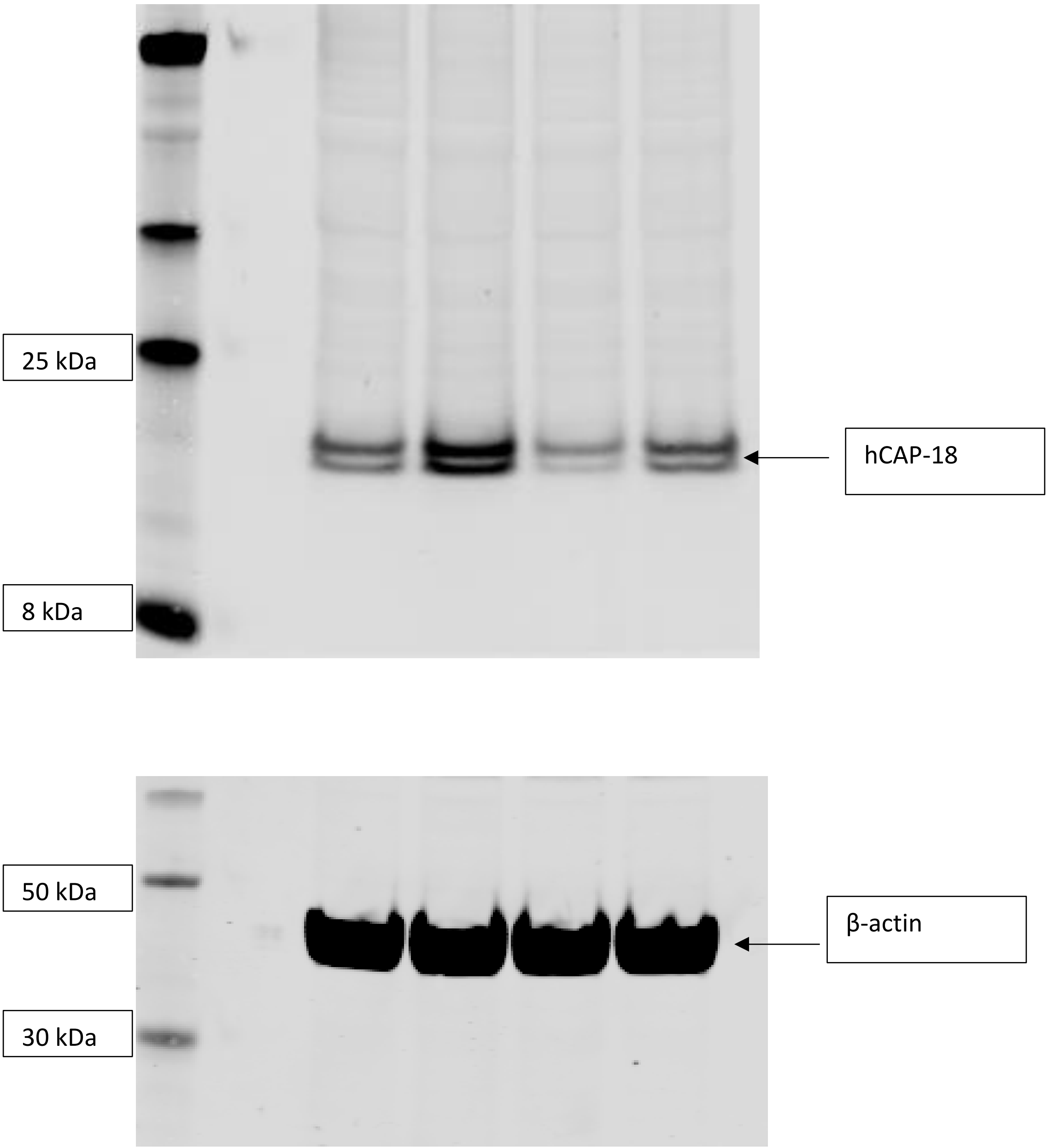

Figure 6D

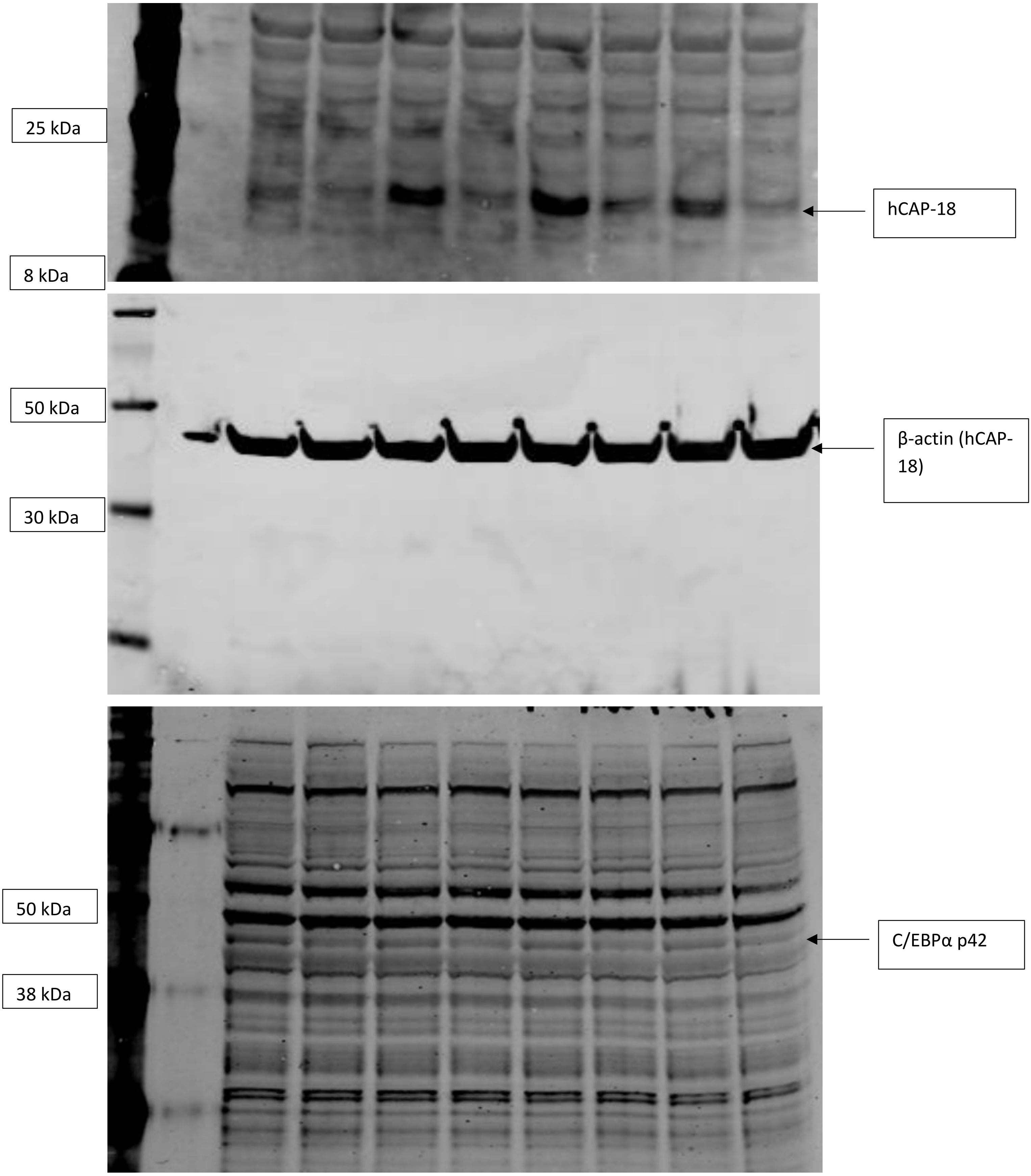
25 kDa
8 kDa
hCAP-18
50 kDa
30 kDa
β-actin (hCAP-18)
50 kDa
38 kDa
C/EBPα p42

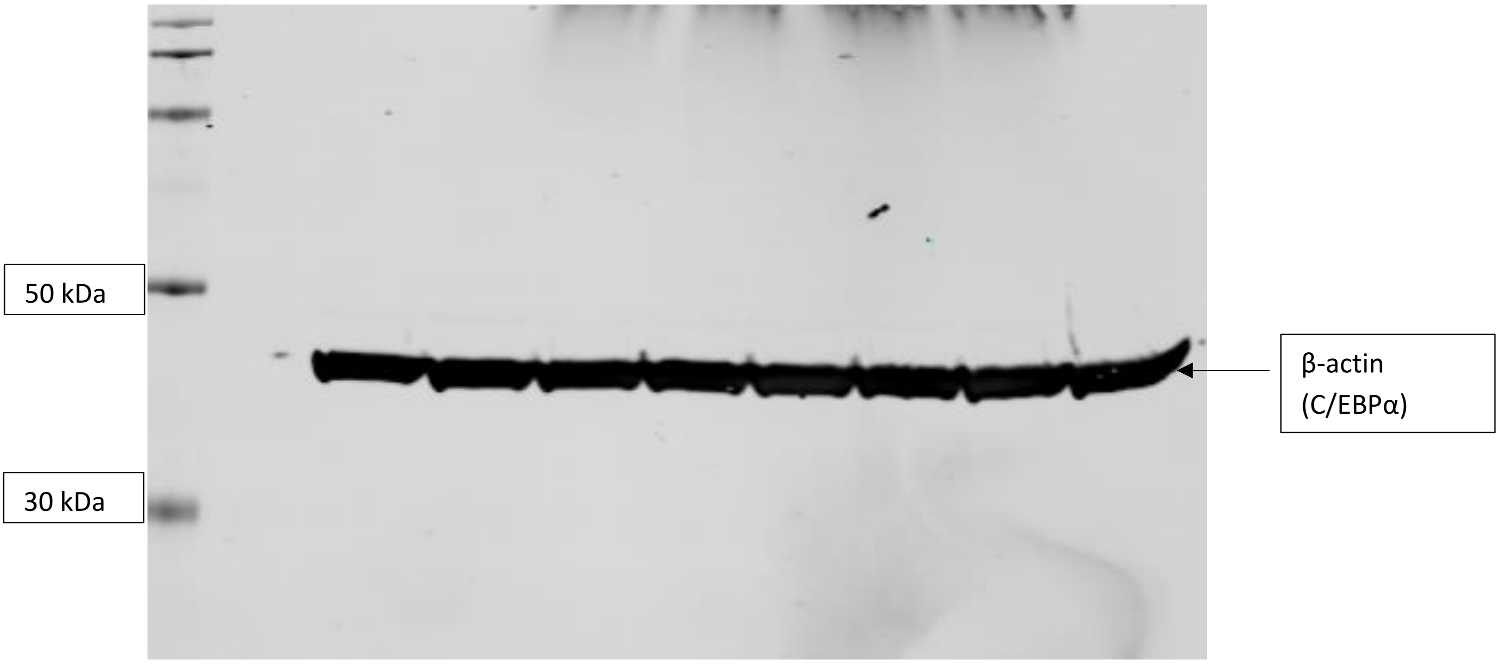
50 kDa
30 kDa
β-actin
(C/EBPα)

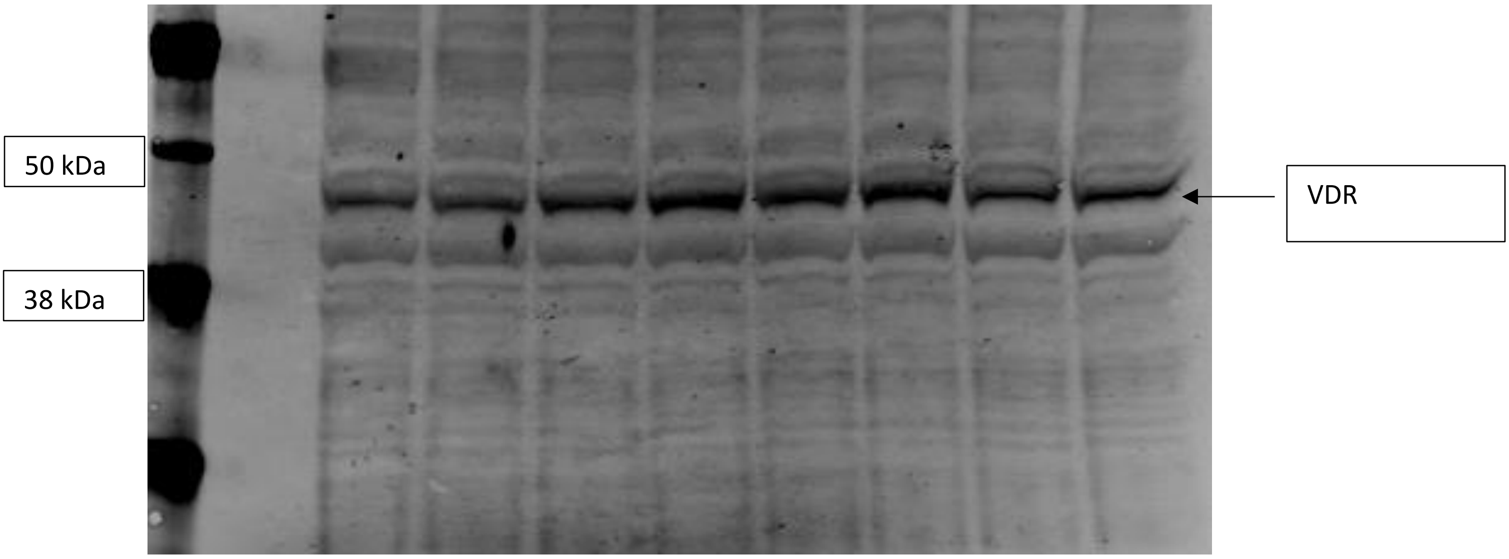
50 kDa
38 kDa
VDR

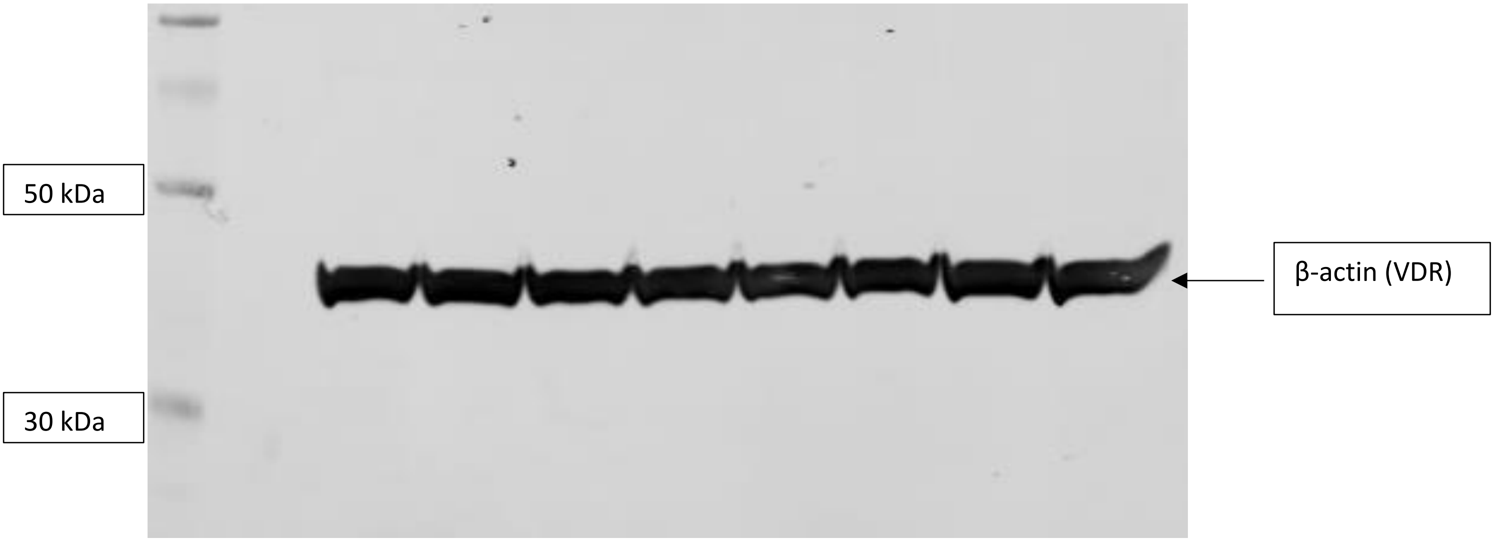

50 kDa
30 kDa
β-actin (VDR)
