# Supplementary material for: Human Metapneumovirus Infection Inhibits Cathelicidin Antimicrobial Peptide Expression in Human Macrophages
Source: Front Immunol. 2018 May 4;9:902. doi: 10.3389/fimmu.2018.00902 (PMC5946005; doi:10.3389/fimmu.2018.00902)
Supplement: Supplementary file 2 [file table_1.PDF]

Supplementary Table 1 – primers for qRT-PCR

Gene	Species	Forward	Reverse
CAMP	human	5´-TCGGATGCTAACCTCTACCG-3´	5´-GTCTGGGTCCCCATCCAT-3´
GAPDH	human	5´-GAAGGTGAAGGTCGGAGTC-3´	5´-GAAGATGGTGATGGGATTTC-3´
CEBPA	human	5’-GGAGCTGAGATCCCGACA-3’	5’-TTCTAAGGACAGGCGTGGAG-3’
IFN-β	human	5´-GCCGCATTGACCATCTATGAGA-3´	5´-GAGATCTTCAGTTTCGGAGGTAAC-3´
CYP27B1	human	5´-CGCAGCTGTATGGGGAGA-3´	5´-CACCTCAAAATGTGTTAGGATCTG-3´
VDR	human	5´-GAAGCTGAACTTGCATGAGGA-3´	5´-GTCCTGGATGGCCTCAATC-3´
IL-28A/B	human	5´-AGGGCCAAAGATGCCTTAG-3´	5´-CAGCTCAGCCTCCAAAGC-3´
IL-29	human	5´-GGGACCTGAGGCTTCTCC-3´	5´-CCAGGACCTTCAGCGTCA-3´
ISG54	human	5´-TGGTGGCAGAAGAGGAAGAT-3´	5´-GTAGGCTGCTCTCCAAGGAA-3´
hMPV	hMPV	5´-CATATAAGCATGCTATATTAAAAGAGTCTC-3´	5´-CCTATTTCTGCAGCATATTTGTAATCAG-3´
